# Electroacupuncture attenuates surgical pain-induced delirium-like behavior in mice *via* remodeling gut microbiota and dendritic spine

**DOI:** 10.3389/fimmu.2022.955581

**Published:** 2022-08-08

**Authors:** Liuyue Yang, Weihua Ding, Yuanlin Dong, Cynthia Chen, Yanru Zeng, Zhangjie Jiang, Shuyuan Gan, Zerong You, Yilin Zhao, Yiying Zhang, Xinghua Ren, Shiyu Wang, Jiajia Dai, Zhong Chen, Shengmei Zhu, Lucy Chen, Shiqian Shen, Jianren Mao, Zhongcong Xie

**Affiliations:** ^1^ College of Pharmaceutical Science, Zhejiang Chinese Medical University, Hangzhou, China; ^2^ Department of Anesthesia, Critical Care and Pain Medicine, Massachusetts General Hospital, Harvard Medical School, Boston, MA, United States; ^3^ Department of Anesthesiology, The First Affiliated Hospital of Zhejiang University, Hangzhou, China

**Keywords:** surgical pain, delirium, gut microbiota, dendritic spine, microglia, electroacupuncture, mice

## Abstract

Surgical pain is associated with delirium in patients, and acupuncture can treat pain. However, whether electroacupuncture can attenuate the surgical pain-associated delirium *via* the gut–brain axis remains unknown. Leveraging a mouse model of foot incision-induced surgical pain and delirium-like behavior, we found that electroacupuncture stimulation at specific acupoints (e.g., DU20+KI1) attenuated both surgical pain and delirium-like behavior in mice. Mechanistically, mice with incision-induced surgical pain and delirium-like behavior showed gut microbiota imbalance, microglia activation in the spinal cord, somatosensory cortex, and hippocampus, as well as an enhanced dendritic spine elimination in cortex revealed by two-photon imaging. The electroacupuncture regimen that alleviated surgical pain and delirium-like behavior in mice also effectively restored the gut microbiota balance, prevented the microglia activation, and reversed the dendritic spine elimination. These data demonstrated a potentially important gut–brain interactive mechanism underlying the surgical pain-induced delirium in mice. Pending further studies, these findings revealed a possible therapeutic approach in preventing and/or treating postoperative delirium by using perioperative electroacupuncture stimulation in patients.

## Introduction

Postoperative delirium, one of the perioperative neurocognitive disorders (1), is associated with adverse effects (2, 3). However, the causes, underlying mechanism, and interventions of postoperative delirium remain largely unclear (2, 3).

Clinical investigations have demonstrated that more than 80% of patients have postoperative pain (4), and pain is associated with delirium (5, 6). However, such association is complicated because analgesics used to treat postoperative pain may cause cognitive dysfunction (7). Therefore, it is crucial to establish an animal study to determine whether surgical pain can cause delirium-like behavior, the underlying mechanism, and the potential targeted interventions.

The Confusion Assessment Method (CAM) is a valid and widely utilized clinical instrument used to detect the presence of delirium in patients (8), consisting of four clinical features: (1) acute onset and fluctuating course, (2) inattention, (3) disorganized thinking, and (4) altered level of consciousness. These features occur naturally without learning. Therefore, we developed an animal behavioral test battery to determine delirium-like behaviors in mice, which incorporates natural behaviors (buried food and an open field test) to probe for attention and consciousness and learned behaviors (Y maze test) to assess for cognition. We fully acknowledge the difficulty with modeling delirium in mice, and that no animal model can be equated with human delirium. However, the model can capture domains of delirium analogously, including attention, cognition, and consciousness, and thus will help us to investigate the pathogenesis of delirium. Employing the model, our previous studies demonstrated that anesthesia/surgery caused an age-dependent delirium-like behavior in mice (9, 10). However, the effects of surgical pain on delirium-like behavior in mice have not been investigated.

Gut microbiota contains up to 95% of the entire human microbiota (11). Gut microbiota dysbiosis, a condition of the altered gut microbiome with an imbalance in unhealthy microorganisms relative to the healthy microorganisms, could lead to altered immune functions and increased risk of diseases (12). Gut microbiota, e.g., *Clostridium tyrobutyricum* and *Akkermansia muciniphila* (13, 14), play a vital role in neurovascular integrity and blood–brain barrier permeability. However, the effects of surgical pain on gut microbiota and the role of gut microbiota in the surgical pain-induced delirium-like behavior remain largely unknown.

Microglia activation is associated with postoperative pain and cognitive deficits (15). Altered synaptic plasticity and synapse loss are associated with cognitive impairment (16, 17). Therefore, we assessed the effects of surgical pain on microglia density and dendritic spine turnover (e.g., formation and elimination).

More importantly, electroacupuncture (EA) stimulation is widely used for pain management (18) and treatment of cognitive impairment induced by stroke, Alzheimer’s disease (AD), and dementia (19, 20). EA inhibits neuroinflammation through remodulating gut microbiome in a mouse model of Parkinson’s disease (21). A recent study demonstrated that EA affects the vagal–adrenal axis in mice (22). Therefore, we set out to test a hypothesis that EA stimulation in specific acupoints can attenuate the surgical pain-induced changes in behavior, gut microbiota, microglia activation, and dendritic spine in mice.

## Methods

### Animals

Adult male and female C57BL/6 (12 to 16 weeks old) mice were purchased from the Jackson Laboratory (USA). C57BL/6 mice (12 to 16 weeks old) expressing YFP in layer V pyramidal neurons (H-line) were purchased from the Jackson Laboratory and group-housed in an animal facility at the Massachusetts General Hospital (Boston, Massachusetts) following animal care regulations. The Massachusetts General Hospital Institutional Animal Care and Use Committee approved the study (protocol 2006N000219). The same mice received both pain threshold and delirium-like behavior tests in the present study.

### Foot incisional pain surgery

The procedure for plantar incision was performed as described previously (23). Briefly, mice were deeply anesthetized with 1.5%–2% isoflurane delivered through a customized nose cone. After the left hind paw was antiseptically prepared with 10% povidone-iodine solution (Medline Industries Inc., Northfield, IL), a 0.5- to 0.8-cm longitudinal incision started 2 mm from the proximal edge of the heel and extended toward the toes was made with a No. 12 curved stainless blade through the skin and fascia of the plantar foot (**Supplementary Figure 1A**). After the underlying muscle was slightly divided, the plantaris tendon was elevated with curved mini-forceps, and a ~0.2-cm longitudinal incision was gently made on it. The skin was opposed with two 6-0 vicryl sutures (Ethicon, Somerville, NJ).

### Electroacupuncture stimulation

EA used a pair of needles so that the impulses could pass from one needle to another as described in previous studies (24). The negative electrode was attached to wound sites, which are considered the main point, while the positive electrode was attached to a secondary point (25). The positive and negative electrodes were demonstrated in **Figure 6** with a red arrowhead and a black arrowhead, respectively. Compiling evidence indicate that dense-disperse waveforms (100/2 Hz) are the effective paradigm in both clinical and animal studies (26, 27).

The combinations of acupuncture points, including Baihui (DU20) and Yongquan (KI1), Baihui and Fengfu (DU16), Kunlun (BL60) and Yongquan, and Huantiao (GB30) and Zusanli (ST36), were performed (**Supplementary Figure 1B**). The acupoints were determined by previously described studies (28–30). The mouse was slightly anesthetized (**Supplementary Figures 1C**, **D**) with isoflurane during EA as described before (31). Positive and negative poles were applied with a pulse stimulator (Model KWD-8081; Ying Di, Guangdong, China) *via* stainless-steel acupuncture needles of 0.2 mm in diameter. The frequency of EA stimulation was 2/100 Hz alternating (2 and 100 Hz shifting automatically). The current intensity was set to induce slight twitches in the hind limbs or body of the mice at 1, 1.5, and 2 mA stepwise for 10 min each, comprising a single 30-min stimulation session (**Supplementary Figure 1E**) following the foot incision surgery in surgical pain (SP) plus EA groups. Furthermore, a dense-disperse waveform was applied for the simulation. Mice in the EA, SP, or SP plus EA group all received the same duration of anesthesia (1.4% isoflurane for 60 min). The mice in the sham group received neither SP nor EA. EA stimulation was performed immediately following incision in the SP plus EA group. The mice in the EA group received 30 min of EA. The mice in the SP plus EA group received 30 min of EA stimulation immediately after SP. We only assessed the EA-induced antinociceptive effect at 2 and 12 h after the surgical pain because we focused on the determination of the acute effects of EA on pain.

### Mechanical withdrawal threshold

A mouse was placed on an elevated ironic mesh platform and was covered with a transparent Plexiglas chamber (10 cm × 10 cm × 12 cm). After ~30 min of acclimation, withdrawal responses to punctate mechanical stimuli were determined using von Frey filaments (**Supplementary Figure 1F**). Each monofilament was applied five times to the plantar aspect of the left hind paw that underwent surgery for approximately 1 s with a 10-s interval between each stimulation. Monofilament started with a force of 0.04 g and continued ascending order up to 2 g. A positive withdrawal response was defined as the stimulation-related withdrawal of the tested paw. The paw withdrawal threshold (PWT) was defined as the force at which withdrawal occurred at least three out of 5 applications, and 2 g was recorded as the PWT if less than three responses to all filaments occurred.

### Hargreaves test

Thermal hyperalgesia to heat stimulation was evaluated following Hargreaves procedure (32) with minor modification for the mice habituation. Mice were placed on a preheated glass platform (28–29°C) and transparent Plexiglas cubicles for 20–30 min of acclimatization. A radiant heat source emitted from underneath the glass and focused to the middle of the paw underwent incision surgery (**Supplementary Figure 1G**). Paw withdrawal latency was defined as the time (seconds) from the initiation of heat exposure to the hind paw withdrawal. A cutoff time was set at 20 s to avoid tissue damage.

### Open field test

Mice were acclimated for ~30 min before the test. A 40 cm × 40 cm box of the enclosure was applied, and two areas were included: the center zone (20 cm × 20 cm) and the surrounding area. The movement parameters of the mouse were monitored and analyzed *via* a video camera (**Supplementary Figure 1H**) connected to the Any-Maze animal tracking system software (Stoelting Co., Wood Dale, IL). Mouse was individually placed in the middle of the center zone, and parameters like latency to the center arena, time spent in the center zone, and freezing time were analyzed. The duration of open field test was 5 min.

### Y maze test

The Y maze test evaluated spontaneous alternations and short-term spatial memory performance in mice. The Y maze apparatus consisted of three arms at 120° angle and connected at a central zone. Mice were acclimatized for 5–10 min before the training was performed. A video camera was mounted vertically on top of the Y maze, and the live location of the tested mouse was recorded by Any-Maze animal tracking system software (Stoelting Co.). The number of entries to the novel arm and time spent in the novel arm were calculated.

### Buried food test

The buried food test was performed to assess the degree of alertness and consciousness in the animals as described previously (33) with minor modification (9). Mice were fed with sweetened cereal 2 days before the test. After acclimation, the testing mouse was placed into a clean cage with a sweetened cereal pellet buried in the bedding with a thickness of 3–5 cm. The latency of the mouse to eat the food was recorded.

### Calculation of the composite *Z*-score

For a comprehensive assessment of the delirium-related parameters, we used the formula *Z* = [Δ XPain− MEAN(Δ X)Sham]/SD(Δ X)Sham as previously described (9, 10) to calculate the *Z*-score. Briefly, (ΔX)Sham was the change score of mice calculated by the test score in the sham group the score at the baseline; ΔXPain was the change score of mice calculated by the test score in the SP group minus the score at baseline; MEAN(ΔX)Sham was the mean of ΔXSham, and SD(ΔX)Sham was the standard deviation of ΔXSham. The composite *Z* score for the mouse was calculated as the sum of the values of six *Z* scores (latency to eat food, time spent in the center, latency to the center, freezing time, entries in novel arm, and duration in novel arm) normalized with the SD for that sum in the sham conditions. Given that the reduction (rather than increase) in time spent in the center (open field test) and the reduction in duration and entries in the novel arm (Y maze test) indicate impairment of the behavior, we multiplied the *Z* score values representing these behaviors by −1 prior to calculating the composite *Z* score using these values.

### DNA extraction and quantification of fecal bacteria

Mice fresh fecal pellets were collected at 9 h after the procedure of sham, SP, EA, or SP plus EA. Fresh fecal pellets were collected by holding the mouse in one hand, during which the mouse defecated directly in a 2-ml clean tube held in the other hand. One to two fresh fecal pellets from each mouse were collected. According to the manufacturer’s instructions, the microbial community DNA was extracted with the DNA Stool Kit (Magen, China). DNA was quantified with a Qubit Fluorometer using the Qubit dsDNA BR Assay Kit (Invitrogen, USA), and quality was measured by running an aliquot on 1% gel. The abundance of eubacteria in feces was measured by qPCR using a StepOnePlus instrument (Applied Biosystems) with the fecal DNA and 16S rRNA gene primers for eubacteria. The sequences of the primers are 515F (5’-GTGCCAGCMGCCGCGGTAA-3’) and 806R (5’-GGACTACHVGGGTWTCTAAT-3’). The real-time PCR program started with an initial step at 95°C for 3 min followed by 30 cycles of 95°C for 45 s, 56°C for 45 s, 72°C for 45 s, and final extension for 10 min at 72°C for 10 min. The PCR products were purified using Agencourt AMPure XP beads and eluted in an Elution buffer. Libraries were qualified by the Agilent Technologies 2100 bioanalyzer. The validated libraries were used for sequencing on the Illumina HiSeq 2500 platform of BGI America (Cambridge, MA), following the standard pipelines of Illumina and generating 2 × 250 bp paired-end reads. 16S rRNA gene sequencing and data analysis were performed by BGI America (Cambridge, MA). Briefly, after amplicon-based sequencing, OTUs were clustered using Ribosomal Database Project (RDP) Classifier v.2.2 with a 60% threshold and trained on the Greengenes database v201305 by QIIME v1.8.0. Alpha and beta diversity were estimated by MOthur (v1.31.2) and QIIME (v1.8.0) at the OUT level, respectively. Barplot and heatmap of different classification levels for **Figure 3** were plotted with the R package v3.4.1 and the R package plots, respectively.

### Craniotomy

The mouse was placed in an induction chamber and anesthetized with 2%–3% isoflurane followed by 1.4% isoflurane for maintenance during the craniotomy surgery. The cranial window over the somatosensory cortex was created on the contralateral side of the plantar incision. Ophthalmic ointment was applied to prevent corneal from drying after the mouse was loaded into the stereotaxic apparatus. The skin over the dorsal skull was prepared with 10% povidone-iodine solution (Medline Industries Inc., Northfield, IL) and 70% (v/v) isopropyl alcohol swab (BD, Franklin Lakes, NJ). After removing the scalp on the dorsal skull, a bone flap in 5 mm diameter was removed using a dental drill (CellPoint Scientific, Gaithersburg, MD). A coverslip was placed over the surface of the cortex and fastened with dental cement following the method described previously (34).

### *In vivo* transcranial two-photon imaging

After 14 days of recovery, YFP mice that underwent craniotomy were acclimated under a two-photon microscope with head restrained for three consecutive days. The baseline of *in vivo* two-photon imaging was acquired 12 h before the plantar incision using a two-photon microscope equipped with a Mai tai laser (Spectra Physics, KMC 100). The wavelength was set at 910 nm, and the average laser power was ∼25 mV for imaging the dendritic spine. A 20×, 1.0 NA water-immersion objective (Olympus, Japan) was used for imaging dendrites with 4× digital zoom, and Prairie View Software was used for image acquisition. All dendrite views were imaged with Z stacks, repeated dendrite imaging was performed at 6 h and 9 h individually after the plantar incision and/or EA, and the same views of dendrites were imaged.

### Image data analysis

The spine elimination and formation analysis were performed as described previously (35). Briefly, ImageJ (NIH) software was used to analyze all the image stacks. The same dendritic segments were identified from three-dimensional stacks acquired from different time points. The total amount of spines was pooled from the dendritic segments of each mouse. Three-dimensional stacks were created to ensure that body movements and rotation between imaging intervals did not influence spine scanning. Spines were considered the same between imaging views if their positions remained the same distance from adjacent landmarks. Based on the first imaging view, the spine was deemed to be different if they were more than 0.7 μm away from their expected positions. Two-way ANOVA was carried out to determine significant differences between groups.

### Iba1 staining

Immediately after the behavioral tests, mice were anesthetized and transcardially perfused with ice-cold PBS followed by 4% paraformaldehyde. Brains were extracted, immersed in 4% PFA, and stored at 4°C for 48 h. After being washed in PBS, brain hemispheres were cut in 40-µm slices using a Leica vibratome (VT 1000s). Free-floating sections throughout the hippocampus were rinsed in PBS and then incubated with 0.03% Tween-20, 5% BSA, and primary antibody (Iba1; 1:1,000; Wako) in PBS at 4°C for 12 h. Sections were then washed and incubated with anti-rabbit Alexa488 (1:2,000; Invitrogen) for 1 h at room temperature. Images were acquired with a confocal microscope (Nikon, Japan), and images were taken in ×4 and ×20 magnification. The acquired images were analyzed using ImageJ (NIH opensource software).

### Statistical analyses

All the data were expressed as mean ± SEM. Based on our previous studies, sufficient power to detect a significant effect should be achieved using 10–12 mice per group for the behavioral experiments, 4 mice per group for the immunostaining analyses, 3 mice per group for the gut microbiota changing analyses, and 4 mice per group for cortical spine analyses. Based on mechanical withdrawal threshold and thermal paw withdrawal latency tests, the difference in pain behaviors was analyzed using a repeated-measures two-way analysis of variance (ANOVA). Post-hoc comparisons with Bonferroni corrections were used to compare thresholds across groups at indicated time points. Paw deformation count was compared across groups using Fisher’s exact test. One-way ANOVA followed by Bonferroni post-hoc analyses was used for all delirium-like behaviors analyzing. The difference in gut microbiota changing among groups was compared using one-way ANOVA followed by Bonferroni post-hoc analyses. Two-way ANOVA followed by Bonferroni post-hoc analyses were used for the different comparisons in cortical spine formation or elimination among groups. *p* < 0.05 was considered statistically significant, and significance testing was two-tailed in a two-group comparison. For Bonferroni corrections, the adjusted *p*-values, calculated *via* dividing *p*-values by sample size, are reported. Statistical analysis was conducted using GraphPad Prism software (version 8.0).

## Results

### EA stimulation with selective acupoints ameliorated surgical pain in mice

**Figure 1:**
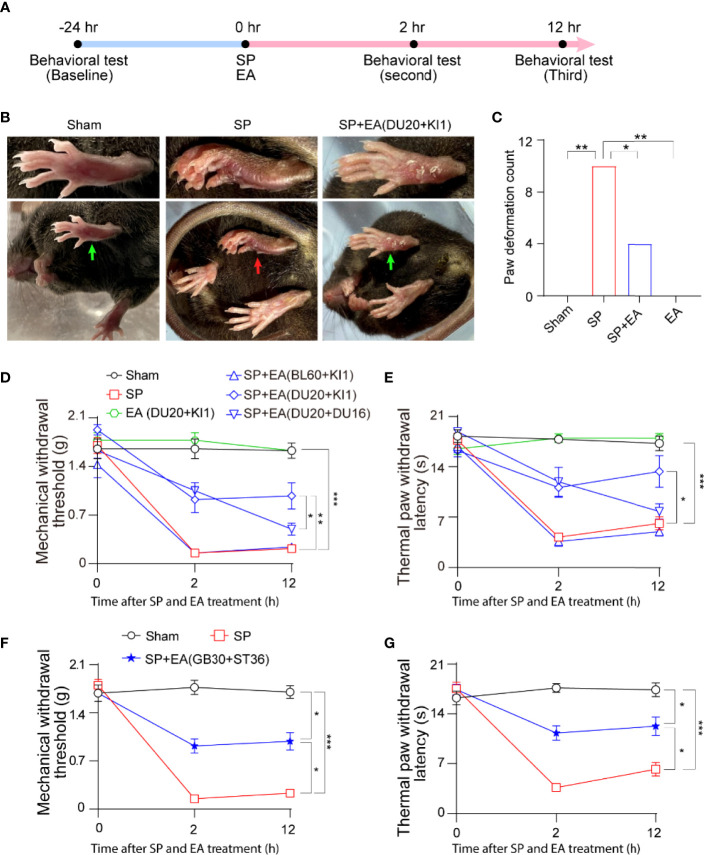
EA attenuates surgical pain (SP) in mice. **(A)** Diagram of procedures and behavioral tests. **(B)** Representative photos of paw deformation induced by SP in the foot. The red arrow indicates paw deformation in surgery mice; green arrows indicate normal paw in sham and SP plus EA mice. **(C)** Paw deformation counted at 12 h after the surgical incision. Effects of EA on paw deformation counted in mice that underwent foot incision. *p*-values indicate the difference in the counts between SP vs. sham; SP vs. SP plus EA (DU20+KI1); SP vs. EA (*n* = 12, Fisher’s exact test). **(D)** Effects of EA on mechanical withdrawal threshold. *p*-values indicate the difference in the threshold between SP vs. sham; SP vs. SP plus EA (DU20+KI1); SP plus EA (DU20+DU16) vs. SP plus EA (DU20+KI1) (*n* = 12, two-way ANOVA followed by Bonferroni post-hoc analyses). **(E)** Effects of EA on thermal paw withdrawal latency. *p*-values indicate the difference in the latency between SP vs. sham; SP vs. SP plus EA (DU20+KI1) (*n* = 12, two-way ANOVA followed by Bonferroni post-hoc analyses). **(F)** Effects of EA on mechanical withdrawal threshold. *p*-values indicate the difference in the threshold between SP vs. sham; SP vs. SP plus EA (GB30+ST36); SP plus EA (GB30+ST36) vs. sham (*n* = 12, two-way ANOVA followed by Bonferroni post-hoc analyses). **(G)** Effects of EA on thermal paw withdrawal latency. *p*-values indicate the difference in the latency between SP vs. sham; SP vs. EA (GB30+ST36); EA (GB30+ST36) vs. sham (*n* = 12, two-way ANOVA followed by Bonferroni post-hoc analyses). Data are represented as mean ± SEM. *p < 0.05, **p < 0.01, ***p < 0.001. SP, surgical pain; EA, electroacupuncture; SEM, standard error of mean; BL60, DU20, DU16, KI1, GB30, and ST36: acupoint.

We set up to determine the interaction of surgical pain (SP) and EA stimulation in pain and behavioral changes in mice (**Figure 1A**). Postural changes in paws have been associated with pain in rodents after hindlimb injury (36). We found that the mice with SP by plantar incision showed significant postural changes in hind paw, as evidenced by the appearance of ventroflexed toes and everted paw, compared to those with sham condition (**Figure 1B**). Quantification of the hind paw deformation demonstrated that EA stimulation itself did not significantly change the count of paw deformation. However, the EA stimulation at DU20+KI1, but not BL60+KI1 (data not shown), acupoint attenuated the SP-induced increase in the count of deformation (**Figure 1C**, *p* < 0.01, Fisher’s exact test).

Next, the SP decreased the mechanical withdrawal threshold (**Figure 1D**) and thermal paw withdrawal latency (**Figure 1E**) compared to the sham condition. The EA stimulation at acupoint DU20+KI1, but not BL60+KI1 or DU20+DU16, attenuated the SP-induced reduction in mechanical withdrawal threshold (**Figure 1D**) and reduction in thermal paw withdrawal latency (**Figure 1E**). Moreover, we found that EA stimulation at GB30+ST36 acupoint also mitigated the SP-induced decreases in mechanical withdrawal threshold (**Figure 1F**) and decreases in thermal paw withdrawal latency (**Figure 1G**). These data demonstrated that plantar incisions could cause SP in mice, and EA stimulation with selective acupoints attenuated the SP in the mice.

### SP-induced delirium-like behavior in mice and EA stimulation attenuated it

**Figure 2:**
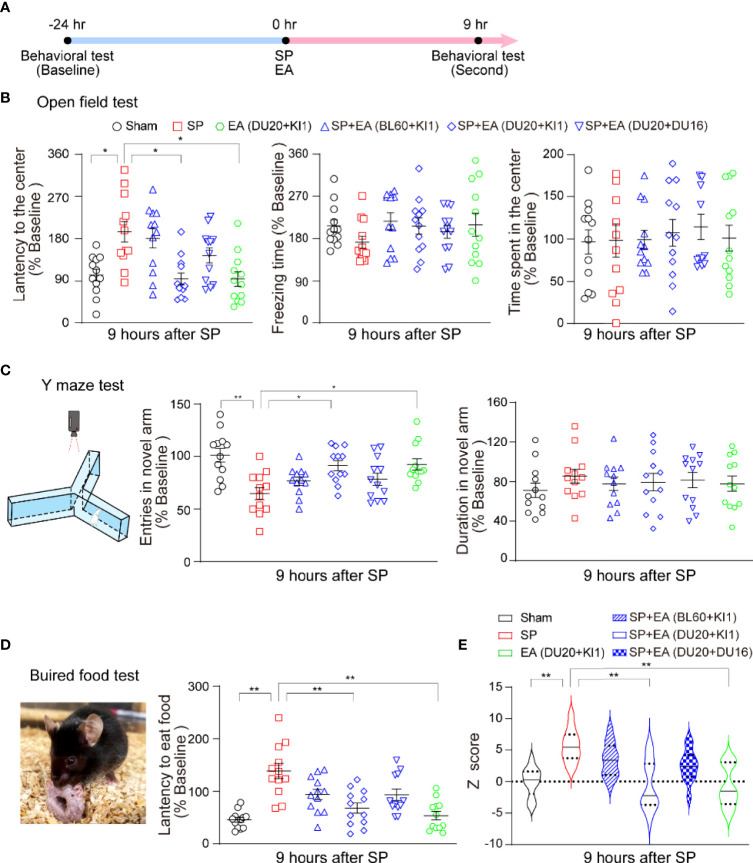
EA attenuates the SP-induced delirium-like behavior in mice. **(A)** Diagram of procedures and behavioral tests. **(B)** Effects of EA and SP on latency to the center (left panel), freezing time (middle panel), and the time spent in the center (right panel) of the open field test. *p*-values indicate the difference in these changes between SP vs. sham; SP vs. SP plus EA (DU20+KI1); SP vs. EA (DU20+KI1) (*n* = 12, one-way ANOVA followed by Bonferroni post-hoc analyses). **(C)** The carton of the Y maze test (left panel). The effects of EA and SP on entries in the novel arm (middle panel) and duration in the novel arm (right panel) of Y maze test in mice at 9 h after the surgery. *p*-values indicate the difference in these changes between SP vs. sham; SP vs. SP plus EA (DU20+KI1); SP vs. EA (DU20+KI1) (*n* = 12, one-way ANOVA followed by Bonferroni post-hoc analyses). **(D)** The picture of the buried food test (left panel). The effects of EA and SP on the latency to eat food in the mice at 9 h after the surgery (right panel). The *p*-values indicate the difference in the latency between SP vs. sham, SP vs. SP plus EA (DU20+KI1), SP vs. EA (DU20+KI1) (*n* = 12, one-way ANOVA followed by Bonferroni post-hoc analyses). **(E)** The effects of EA and SP on composite *Z* score in mice at 9 h after the surgery. EA (DU20+KI1) attenuated the SP-induced delirium-like behavior in the mice. *p*-values indicate the difference in the composite *Z* score between SP vs. sham; SP vs. SP plus EA (DU20+KI1); SP vs. EA (DU20+KI1) (*n* = 12, one-way ANOVA followed by Bonferroni post-hoc analyses). Data are represented as mean ± SEM. *p < 0.05, **p < 0.01. SP, surgical pain; EA, electroacupuncture; SEM, standard error of mean; BL60, DU20, DU16, KI1: acupoint.

Next, we used our established methods to determine the interaction of SP and EA stimulation on delirium-like behavior in mice at 9 h after the SP (**Figure 2A**). We found that the SP induced changes in the latency to the center, freezing time and time in the center of open field test (**Figure 2B**), entries in novel arm and duration in the novel arm of the Y maze test (**Figure 2C**), and the latency to eat food in the buried food test (**Figure 2D**) compared to the sham condition. EA stimulation itself did not significantly affect these changes, but EA stimulation at DU20+KI1 mitigated these SP-induced changes in the mice. More importantly, SP increased the composite *Z* score compared to the sham condition in mice (**Figure 2E**). The EA stimulation with acupoints DU20+KI1, but not DU20+DU16 and BL60+KI1, attenuated the SP-induced increases in the composite *Z* score in mice (**Figure 2E**). Taken together, these data suggest a potential protective effect of EA stimulation with selective acupoints (e.g., DU20+KI1) on the SP-induced delirium-like behavior in mice.

### SP induced gut microbiota dysbiosis and EA stimulation restored it

**Figure 3:**
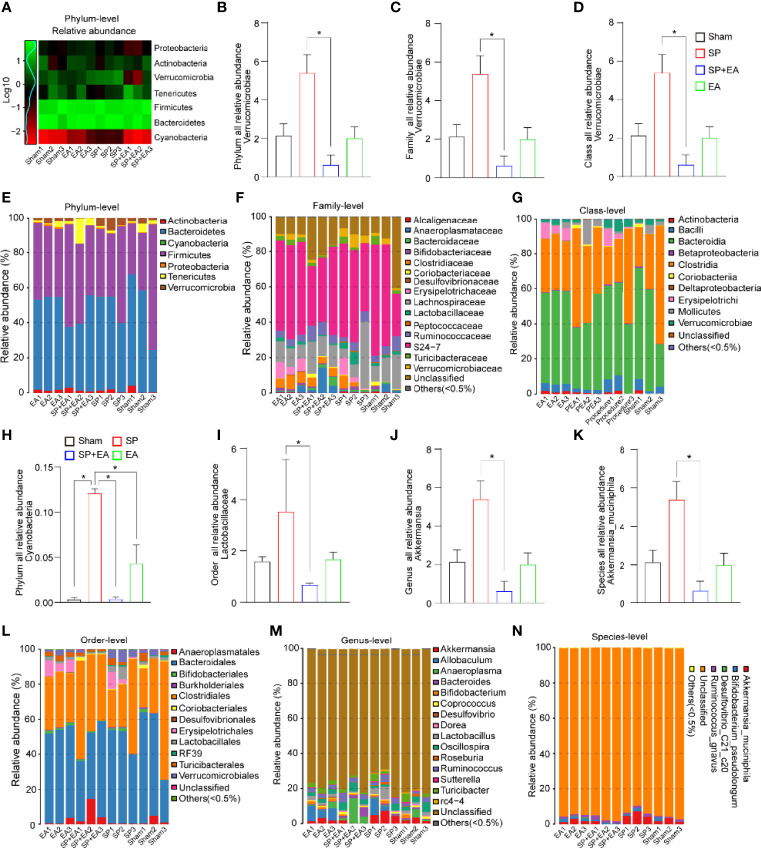
EA (DU20+KI1) attenuates the SP-induced changes in gut microbiota in mice. **(A)** Log-scaled percentage heatmap of phylum level. **(B-G)** SP increased the abundance of *Verrucomicrobia* at the phylum, family, and class levels. EA mitigated such increases. The *p*-values indicate the difference in the abundance between SP and SP plus EA (*n* = 3, one-way ANOVA followed by Bonferroni post-hoc analyses). **(H)** SP increased the abundance of *Cyanobacteria* at the phylum level indicated in **(E)**. EA mitigated such increases. The *p*-values indicate the difference in the abundance between SP and SP plus EA (*n* = 3, one-way ANOVA followed by Bonferroni post-hoc analyses). **(I–L)** SP increased the abundance of *Lactobacillales* at the order level. EA mitigated such increases. The *p*-values indicate the difference in the abundance between SP and SP plus EA (*n* = 3, one-way ANOVA followed by Bonferroni post-hoc analyses). **(J, K, M, N)** The taxonomic composition distribution in samples at genus and species level. SP increased the abundance of *Akkermansia* at the genus level. EA mitigated such increases. The *p*-values indicate the difference in the abundance between SP and SP plus EA (*n* = 3, one-way ANOVA followed by Bonferroni post-hoc analyses). Data are represented as mean ± SEM. *p < 0.05. SP, surgical pain; EA, electroacupuncture; SEM, standard error of mean.

Given that we already identified DU20 + KI1 as the effective acupoint, the action of which by EA mitigated the SP-induced pain and delirium-like behavior in mice, next, we focused on DU20 + KI1 in the mechanistic studies. Changes in gut microbiota at phylum, family, order, class, genus, and species levels were observed in the mice following SP compared to the sham condition. Specifically, the SP increased *Verrucomicrobia* abundance at phylum, family, and class levels in the mice compared to the sham condition (**Figures 3A**–**G**). EA stimulation at DU20+KI1 significantly mitigated the SP-induced changes in *Verrucomicrobia* abundance at phylum (**Figures 3B**, **E**), family (**Figures 3C**, **F**), and class levels (**Figures 3D**, **G**). The *Cyanobacteria* abundance was increased at the phylum level (**Figure 3H**), and the *Lactobacillaceae* abundance was increased at the order level (**Figure 3I, L**) following SP, and EA stimulation at DU20+KI1 significantly mitigated such increases at the order levels. Furthermore, the SP increased *Akkermansia* abundance at genus and species levels, and EA stimulation attenuated such increases (**Figures 3J**, **K**, **M**, **N**). Collectively, these data indicated that EA stimulation was able to prevent, at least partially, the gut microbiome dysbiosis induced by SP in mice.

### EA stimulation mitigated the SP-induced increases in microglia density in mice

**Figure 4:**
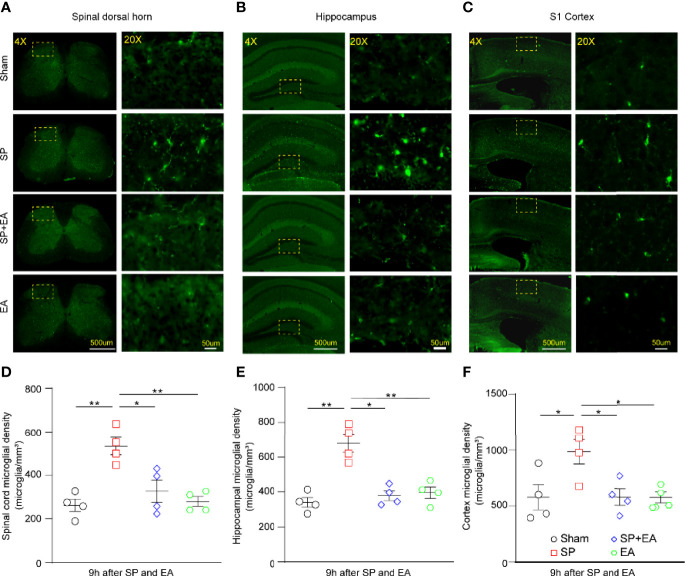
EA (DU20+KI1) attenuates the SP-induced increases in microglia density in the spinal dorsal horn, hippocampus, and somatosensory cortex in mice. **(A–C)** Representative Iba1 staining of sham, SP, SP plus EA, and EA mice were taken at 4× (scale bar represents 500 µm); the boxed regions were zoomed-in (20×) with scale bar representing 50 µm. **(D)** Spinal cord microglia density significantly increased in mice that underwent plantar surgical incision. *p*-values indicate the difference in the microglia density between SP vs. sham, SP vs. SP plus EA, and SP vs. EA (*n* = 4, one-way ANOVA followed by Bonferroni post-hoc analyses). Data are represented as mean ± SEM. *p < 0.05, **p < 0.01. **(E)** Microglia density upregulated in the hippocampus of mice that underwent plantar surgical incision. *p*-values indicate the difference in the microglia density between SP vs. sham, SP vs. SP plus EA, and SP vs. EA (*n* = 4, one-way ANOVA followed by Bonferroni post-hoc analyses). **(F)** Somatosensory cortex microglia density significantly increased in mice that underwent plantar surgical incision. The *p*-values indicate the difference in the microglia density between SP vs. sham, SP vs. SP plus EA, and SP vs. EA (*n* = 4, one-way ANOVA followed by Bonferroni post-hoc analyses). SP, surgical pain; EA, electroacupuncture; SEM, standard error of mean.

We performed immunohistochemistry using a pan microglia marker (Iba1) to assess the effect of SP and EA stimulation (DU20+KI1) on the changes in microglia density in the spinal cord, somatosensory cortex, and hippocampus of mice. The SP increased microglia density in the spinal dorsal horn (**Figures 4A**, **D**), hippocampus (**Figures 4B**, **E**), and cortex (**Figures 4C**, **F**). EA stimulation (DU20+KI1) significantly attenuated such increases in the mice.

### EA stimulation mitigated the SP-induced changes in the dendritic spine in mice

**Figure 5:**
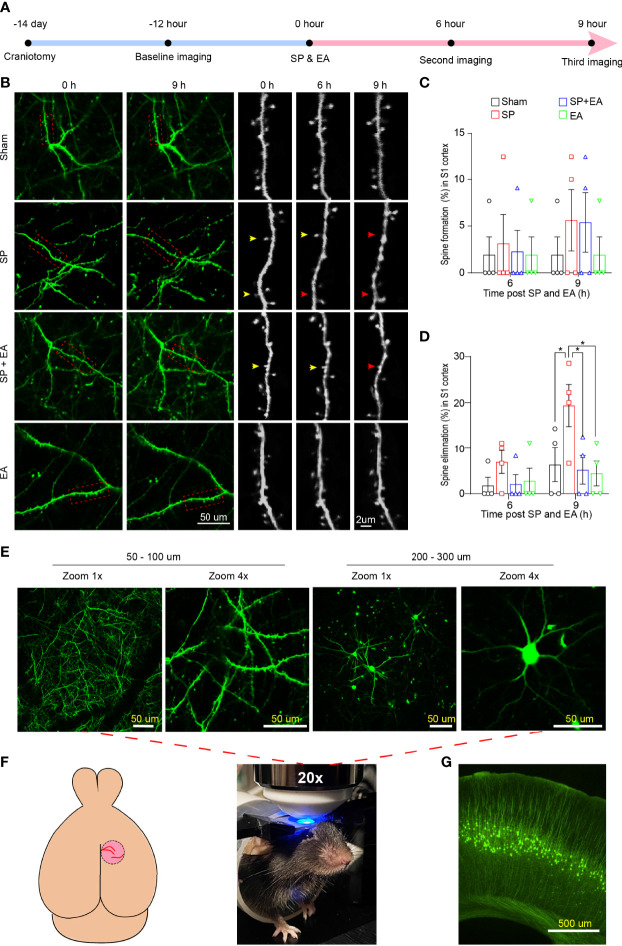
EA (DU20+KI1) attenuates the SP-induced changes in the formation and elimination of the cortical dendritic spine in mice 9 h after surgical incision. **(A)** Diagram of transcranial two-photon imaging workflow. **(B)** Left two panels, representative two-photon images of same pyramidal dendrites acquired at baseline and 9 h after surgical incision individually. Right three panels, repeated imaging of the boxed dendritic segment after surgical incision and EA stimulation with a higher-magnification view of the boxed dendritic segment at baseline, 6 h, and 9 h after the surgical incision. Arrowheads indicate dendritic spines dynamics at the indicated time points. Yellow arrowheads represent spines that remained *in situ*; red arrowheads represent spine elimination. **(C)** SP did not cause significant differences in the dendritic spines formation rate in the somatosensory cortex of mice compared to sham condition at 6 or 9 h after the procedure (*n* = 4, two-way ANOVA followed by Bonferroni post-hoc analyses). **(D)** SP significantly increased the dendritic spines elimination rate in the somatosensory cortex of mice compared to sham condition at 6 or 9 h after the procedure (*n* = 4, two-way ANOVA followed by Bonferroni post-hoc analyses). *p*-values indicate the difference in spine elimination rate between SP vs. sham, SP vs. SP plus EA, and SP vs. EA (*n* = 4, two-way ANOVA followed by Bonferroni post-hoc analyses. **(E)** Lower panel, mouse head restrained for transcranial two-photon imaging; upper panel, representative images of cortical dendrites and pyramidal neurons at different depths of the S1HL cortex in YFP mouse. Scale bars represent 50 µm. **(F)** Schematic of the mouse brain and transcranial window for two-photon imaging. **(G)** Representative YFP mouse brain slice taken at 4×. Scale bar represents 500 µm. All images were scanned with a green filter. Data are represented as mean ± SEM. *p < 0.05. SP, surgical pain; EA, electroacupuncture; SEM, standard error of mean.

To assess the interaction of SP and EA stimulation on dendritic spine turnover, transcranial *in vivo* two-photon imaging was performed in mice (**Figure 5A**). The SP slightly increased the dendritic spine formation in the S1 cortex of mice compared to the sham condition (**Figures 5B**, **C**). However, the SP significantly increased the elimination of the dendritic spine in the S1 cortex of mice compared to the sham condition (**Figures 5B**, **D**).

EA stimulation at acupoints DU20+KI1 attenuated the SP-induced remodeling of the dendritic spine, including formation and elimination, in the mice (**Figures 5C**, **D**). Representive imaging (**Figures 5E**), schematic of the mouse brain transcranial window (**Figures 5F**), and representative YFP mouse brain slice (**Figure 5G**) of two-photon were demonstrated. These data indicated that EA stimulation could attenuate the surgical pain-induced delirium-like behavior, gut microbiota dysbiosis, microglia activation, and dendritic spine remodeling in the mice.

## Discussion

**Figure 6:**
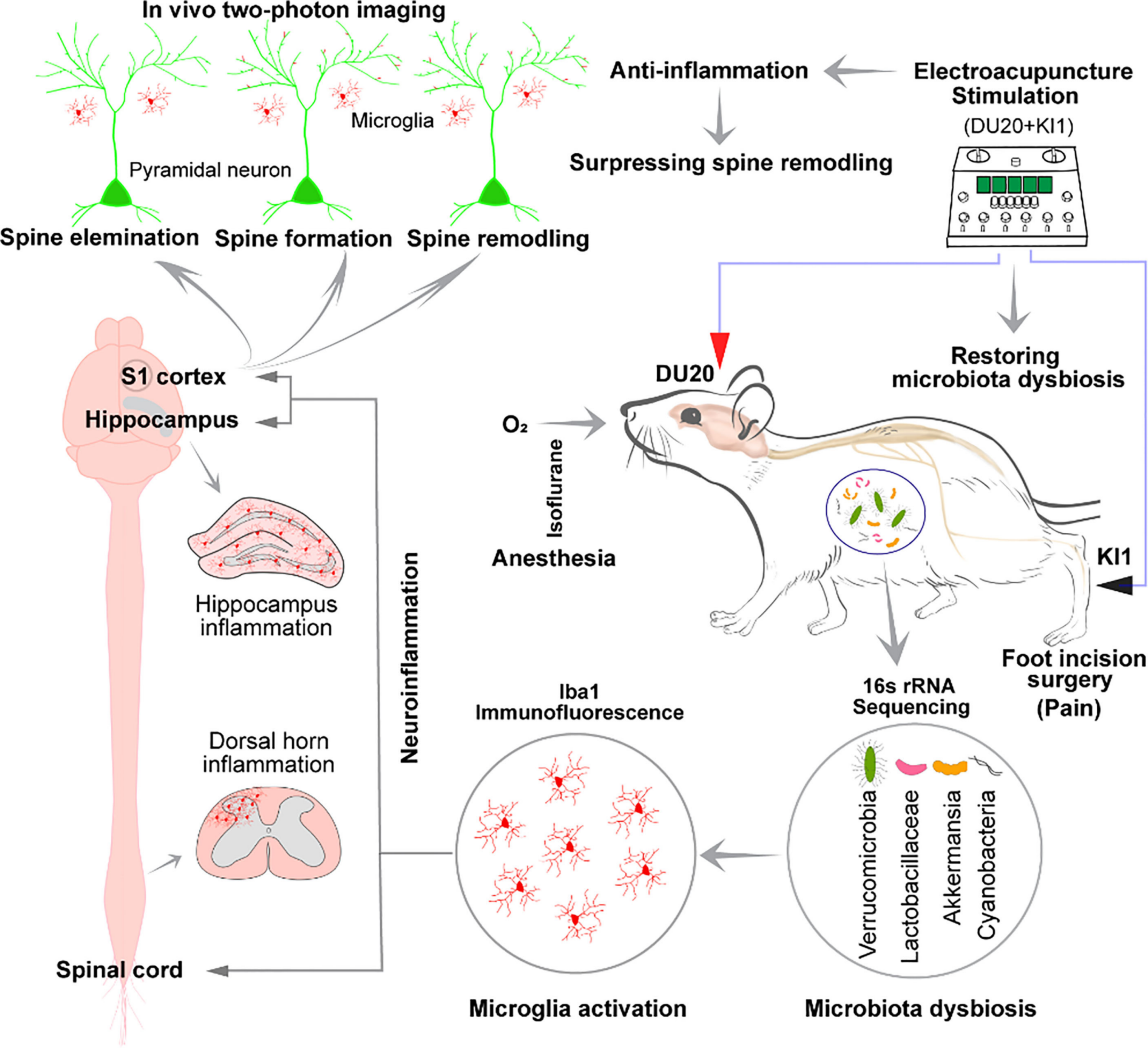
Hypothesized pathway of surgical pain-induced delirium-like behavior. Surgical incision in the foot causes pain and delirium-like behavior in mice; changes in gut microbiota; microglia activation at spinal dorsal horn, hippocampus, and somatosensory cortex in mice; and remodeling of the cortical dendritic spine in mice. EA stimulation at specific acupoints DU20+KI1 ameliorated these changes. Mechanistically, we hypothesize that surgical pain increased microglia activation and dendritic spine remodeling, which are mediated by gut microbiota dysbiosis, leading to delirium-like behavior in mice.

In this proof-of-concept study, we demonstrated that surgical pain (SP) induced gut microbiota dysbiosis, microglia activation, and dynamic changes (e.g., formation and elimination) in the dendritic spine, as well as the delirium-like behavior in mice. More importantly, EA stimulation at specific acupoints (e.g., DU20+KI1 or GB30+ST36) attenuated these SP-induced changes in mice. Pending further studies, these results suggest a working hypothesis that SP induces delirium-like behavior by affecting microglia activation and dendritic spine remodeling mediated by gut microbiota (**Figure 6**). These results also revealed that EA stimulation could prevent or treat the SP-induced delirium-like behavior in mice, as well as the changes in gut microbiota, microglia, and dendritic spine.

Plantar incision surgery is associated with increased spontaneous pain, mechanical allodynia, and thermal hyperalgesia (37, 38). In the present study, we observed an increased number of postoperative paws deformation in mice subjected to the plantar incision, suggesting increased pain in mice following the plantar incision. Moreover, the plantar incision decreased mechanical withdrawal threshold and thermal paw withdrawal latency, further suggesting that the plantar incision caused pain in mice. The EA stimulation at specific acupoints prevented the plantar incision-induced surgical pain, mechanical allodynia, and thermal hyperalgesia. These findings suggest that EA stimulation is a potential therapy for surgical pain, consistent with the data obtained from a previous study (39).

Postoperative pain is associated with severe peripheral inflammation and a central mechanism involving microglia activation at the level of the spinal cord and the brain (37). Elevated levels of pro-inflammatory cytokines are associated with postoperative delirium in patients (40, 41) and delirium-like behavior in animals (15, 42). Consistently, immunohistochemical analysis using a pan microglia marker revealed an increase in the density of microglia in the present study. These data suggest that SP increased microglia activation and neuroinflammation at the spinal cord, hippocampus, and somatosensory cortex in mice. Our results were consistent with the recent finding that surgery alters the morphology of microglia in the clarified hippocampus (43). EA stimulation prevented SP-induced neuroinflammation by reducing the activation of microglia at the spinal cord, somatosensory cortex, and hippocampus. These findings suggest the anti-inflammatory effect of EA stimulation in mice.

SP also could cause gut microbiome dysbiosis at various levels in mice in the present study. Consistently, previous studies showed that gut microbiota dysbiosis was associated with pain (44–47). A recent investigation reported that anesthesia/surgery caused age-dependent changes in the gut microbiota, e.g., increases in the abundance of Lactobacillus and delirium-like behavior in mice (10). Gut microbiota dysbiosis increases blood–brain barrier permeability by modulating the synthesis of short-chain fatty acid (13). The increased blood–brain barrier permeability may exacerbate the neuroinflammation and worsen the neuropathogenesis of delirium (48, 49). In the present study, EA stimulation mitigated the SP-induced gut microbiome dysbiosis and microglia activation in the hippocampus, somatosensory cortex, and spinal cord, the central nervous system regions primarily associated with cognition and anxiety, and pain in mice. In future studies, we will use the established system to determine whether EA stimulation can work at different levels, e.g., site of plantar incision, spinal cord, blood–brain barrier permeability, or brain regions, to mitigate the SP-induced delirium-like behavior and the associated changes.

Dendritic spine turnover is associated with cognitive function (50). In the present study, the SP altered dendritic spine remodeling by significantly increasing the elimination of the dendritic spine in the S1 cortex of the mice. EA stimulation was able to prevent these changes.

The analgesic effect of EA stimulation is well documented (31), and a recent study demonstrated that EA stimulation ameliorated cognitive impairment *via* inhibition of synaptic degeneration and neuroinflammation in a mouse model of AD (51). Stimulation at DU20 and KI1 by EA suppressed brain microglia activation (52, 53). It contributed to neuroprotection against chronic unpredictable mild stress by enhancing brain-derived neurotrophic factor expression and improving hippocampal neurogenesis in a rat model of depression (54). Furthermore, EA stimulation at DU20 and KI1 ameliorated learning and memory deficits and enhanced synaptic plasticity *via* activation of the PKA/CREB signaling pathway (55). Another study indicated that EA stimulation at KI1 significantly prevented spatial learning and memory impairment in rodents *via* the mediation of nicotinamide adenine dinucleotide phosphate (NADPH)-oxidase2 (NOX2) in hippocampus (56). NOX2 has been identified as an upstream factor controlling oxidative stress and neuroinflammation. EA stimulation alleviates behavioral changes *via* modulating gut microbiota and suppressing neuroinflammation in a mouse model of Parkinson’s disease (57). A recent study demonstrated that EA affects the vagal–adrenal axis in mice (22). Consistently, the present study revealed the new effects of EA stimulation on ameliorating pain, neuroinflammation, dendritic spine remodeling, and delirium-like behavior induced by SP in the mice. Nevertheless, these findings may ultimately lead to clinical investigation to determine whether EA stimulation can prevent and treat delirium in patients.

Finally, anesthesia (58) and surgery (59) may reduce synapse number in rodents. A recent study showed that changes in gut microbiota contributed to postoperative cognitive impairment in mice via valeric acid (60). Moreover, low intensity exercise stabilized the surgery-induced changes in gut microbiota (60). Consistently, the findings from the present study also demonstrated that pain could change gut microbiota and acupuncture mitigated the pain-induced changes in microbiota.A recent study has demonstrated that the transcutaneous electrical acupoint stimulation mitigated the postoperative paralytic ileus incidence and enhanced gastrointestinal functional recovery, suggesting that electrical acupoint stimulation could increase parasympathetic nerve tone and decrease inflammation (61) Consistently, the data from the present study showed that electroacupuncture mitigated the pain-induced changes in gut microbiota. The future studies will include the investigation of whether such action can be mediated by changes in parasympathetic nerve tone.

The current study has several limitations. First, there has been no ideal animal model to study delirium-like behaviors. Our previous studies developed an animal behavioral test battery, including natural behavior (buried food and an open-field test) to probe for attention and awareness, and learned behavior (Y maze test) to assess cognition (9, 10). We recognize that no test can fully model delirium in mice, and no animal model of delirium-like behavior equates human delirium. However, the current battery of behavioral tests captured certain domains of delirium-like behavior in mice and can be used for delirium research. We used both female and male mice in the present study without determining the sex-dependent effects. This is because the objective of the present study was not to illustrate the sex-dependent changes in the surgical pain-induced delirium-like behavior but to determine the interaction of surgical pain and EA stimulation on delirium-like behavior and the potential underlying mechanisms. We will use the established model further to identify the possible sex-dependent changes in the future.

In conclusion, the present study demonstrated that SP could induce gut microbiota dysbiosis, microglia activation, remodeling of the dendritic spine, and delirium-like behavior in mice. EA stimulation in specific acupoints prevented these changes. Pending further investigations, the findings from the present study demonstrated the role of SP in delirium-like behavior in mice and suggested the potential application of EA in preventing and treating delirium-like behavior in mice. These findings would promote more research, ultimately leading to clinical investigation to determine whether EA stimulation can prevent or treat postoperative delirium in patients.

## Data availability statement

The sequencing data have been deposited to NCBI Trace Archive NCBI Sequence Read Archive (https://www.ncbi.nlm.nih.gov/sra/PRJNA861064) with the bioproject_accession number: PRJNA861064. The other data will be available from corresponding authors upon reasonable request.

## Ethics statement

This study was reviewed and approved by Massachusetts General Hospital Institutional Animal Care and Use Committee approved the study.

## Author contributions

ZX, SS, and JM planned and designed the experiments. LY, ZY, YiyZ, and YD coordinated all the experiments. LY performed behavioral experiments and immunohistochemical staining. CC, YanZ, ZJ, SG, XR, SW, and JD assisted surgery and behavioral experiments. LY and YilZ carried out imaging experiment. SZ and ZC provided knowledge on rRNA sequence data analysis. LY analyzed the data and presented the figures. LY, CC, and WD drafted the manuscript. LC, SS, JM, and ZX reviewed and revised the manuscript. All authors contributed to the article and approved the submitted version.

## Funding

This research was supported by 1RF1AG070141, 1R21AG065606, and 5R35GM128692 from the National Institutes of Health, Bethesda, MD to SS, and by R01AG041274, R01AG062509, and RF1AG070761 from the National Institutes of Health, Bethesda, MD to ZX.

## Acknowledgments

These studies were performed at the Massachusetts General Hospital and Harvard Medical School and are attributed to the Department of Anesthesia, Critical Care and Pain Medicine, Massachusetts General Hospital, and Harvard Medical School.

## Conflict of interest

The authors declare that the research was conducted in the absence of any commercial or financial relationships that could be construed as a potential conflict of interest.

The handling editor NT declared a past group authorship with the author ZX.

## Publisher’s note

All claims expressed in this article are solely those of the authors and do not necessarily represent those of their affiliated organizations, or those of the publisher, the editors and the reviewers. Any product that may be evaluated in this article, or claim that may be made by its manufacturer, is not guaranteed or endorsed by the publisher.
