# Supplementary material for: Electroacupuncture attenuates surgical pain-induced delirium-like behavior in mice via remodeling gut microbiota and dendritic spine
Source: Front Immunol. 2022 Aug 8;13:955581. doi: 10.3389/fimmu.2022.955581 (PMC9393710; doi:10.3389/fimmu.2022.955581)
Supplement: Supplementary file 1 [file Image_1.pdf]

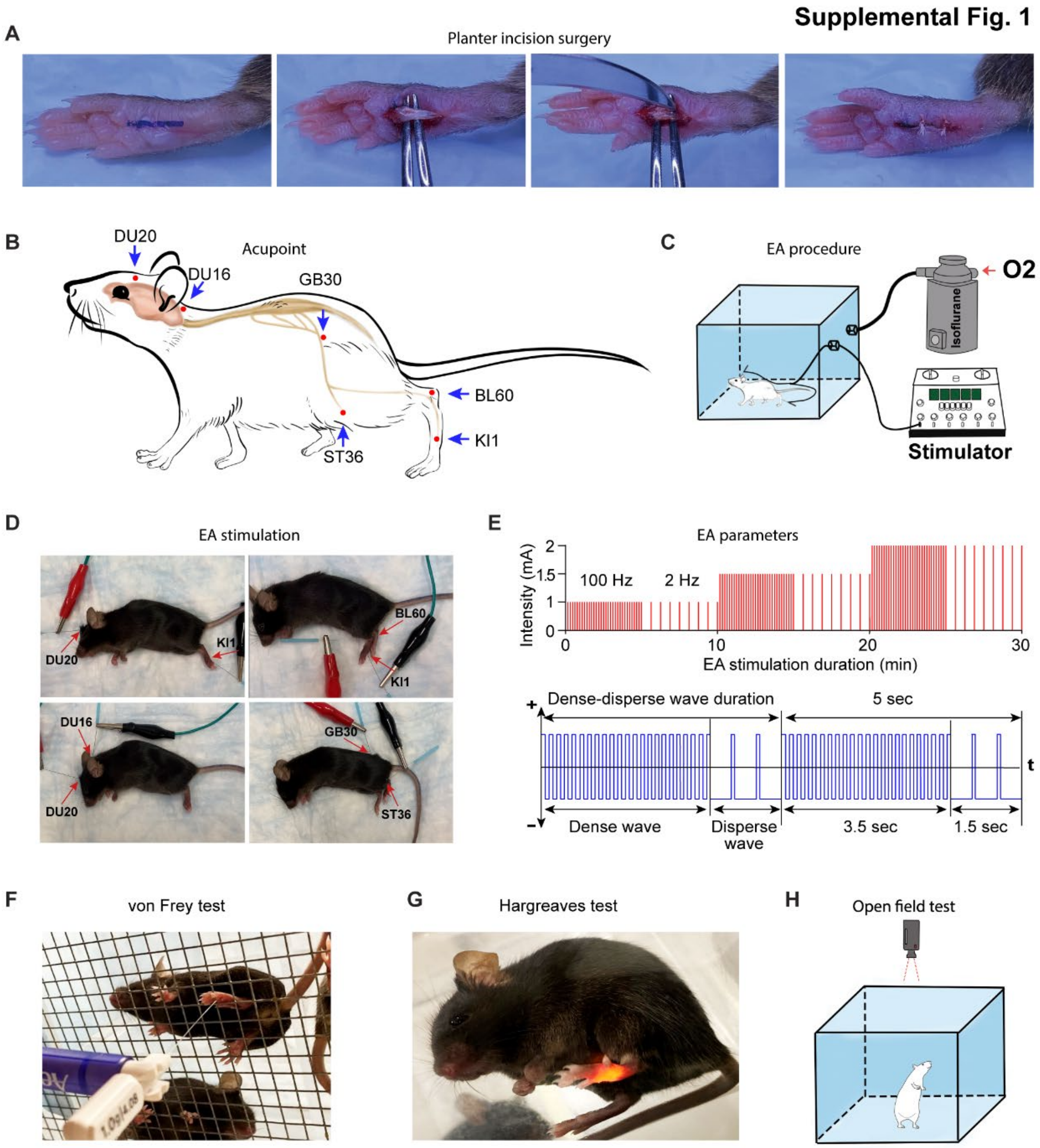

Supplemental Fig. 1. Schematic illustration of experimental design. A) Surgical incision in the foot. The plantar aspect of the left hind paw was prepared, and a longitudinal line was marked for incision. **B)** Schematic of acupoints including DU20, DU16, GB30, ST36, BL60, and KI1 applied in this study. **C)** Schematics of components and devices of EA stimulation for the mouse. **D)** Acupoints combinations were applied in the mouse. Top left, acupoints combination of DU20 and KI1; top right, acupoints combination of BL60 and KI1; bottom left, acupoints combination of DU20 and DU16; bottom right, acupoints combination of GB30 and ST36. **E)** Microcurrent waveforms for EA stimulation. The upper panel represents the intensity of microcurrent for EA stimulation. The frequency of EA stimulation was 2/100 Hz alternating (2 and 100 Hz shifting automatically), and the current intensity was set to 1, 1.5, and 2 mA stepwise for 10 min each, comprising a 30-min stimulation session. Lower panel, the dense-dispersed

waveform was applied for stimulation. **F)** Photo of von Frey test. **G)** Photo of Hargreaves test. **H)** Schematic of the open field test.
